# FEDERAL POLICY EFFORTS TO SUPPORT FAMILY CAREGIVERS: PAST, PRESENT, AND FUTURE

**DOI:** 10.1093/geroni/igz038.493

**Published:** 2019-11-08

**Authors:** Lauren R Bangerter, Nicole Ruggiano, Joan M Griffin, Kelly A O Malley

**Affiliations:** 1 Mayo Clinic, Rochester, United States; 2 University of Alabama, Tuscaloosa, Alabama, United States; 3 New England GRECC, Boston VA Healthcare System, Boston, Massachusetts, United States

## Abstract

Family caregivers are major providers of long-term care and support for family members with age-related decline and disability. Caregiving is a demanding and complex undertaking that has demonstrated extensive physical, emotional, relational, and financial burden on families. These factors underscore the urgency of addressing the needs of family caregivers at the policy level. This presentation focuses on the origins, goals, and mandates of federal policy efforts that support family caregivers. This includes an analysis on how federal support for caregivers has evolved over time, most notably through the National Family Caregiver Support Program, part of the Older Americans Act. It will also discuss the passage of more recent legislation (e.g., RAISE Family caregiver Act and the Grandparents Raising Grandchildren Act) that will contribute to a national strategy to support family caregivers. Moreover, through the discussion of these policies, we will articulate specific areas where gerontology research and public policy can and should intersect in order to optimize the effectiveness of policy efforts to support family caregivers.

